# Comparison of Mortality Outcomes in Acute Myocardial Infarction Patients With or Without Standard Modifiable Cardiovascular Risk Factors

**DOI:** 10.3389/fcvm.2022.876465

**Published:** 2022-04-14

**Authors:** Ching-Hui Sia, Junsuk Ko, Huili Zheng, Andrew Fu-Wah Ho, David Foo, Ling-Li Foo, Patrick Zhan-Yun Lim, Boon Wah Liew, Ping Chai, Tiong-Cheng Yeo, James W. L. Yip, Terrance Chua, Mark Yan-Yee Chan, Jack Wei Chieh Tan, Gemma Figtree, Heerajnarain Bulluck, Derek J. Hausenloy

**Affiliations:** ^1^Department of Cardiology, National University Heart Centre Singapore, Singapore, Singapore; ^2^Yong Loo Lin School of Medicine, National University of Singapore, Singapore, Singapore; ^3^MD Program, Duke-NUS Medical School, Singapore, Singapore; ^4^Health Promotion Board, National Registry of Diseases Office, Singapore, Singapore; ^5^SingHealth Duke-NUS Emergency Medicine Academic Clinical Programme, Singapore, Singapore; ^6^National Heart Research Institute Singapore, National Heart Centre Singapore, Singapore, Singapore; ^7^Pre-hospital and Emergency Care Research Centre, Health Services and Systems Research, Duke-NUS Medical School, Singapore, Singapore; ^8^Tan Tock Seng Hospital, Singapore, Singapore; ^9^Khoo Teck Puat Hospital, Singapore, Singapore; ^10^Changi General Hospital, Singapore, Singapore; ^11^Department of Cardiology, National Heart Centre Singapore, Singapore, Singapore; ^12^Sydney Medical School (Northern), University of Sydney, Sydney, NSW, Australia; ^13^Leeds Teaching Hospital NHS trust, Leeds, United Kingdom; ^14^Cardiovascular and Metabolic Disorders Program, Duke-National University of Singapore Medical School, Singapore, Singapore; ^15^The Hatter Cardiovascular Institute, University College London, London, United Kingdom; ^16^Cardiovascular Research Center, College of Medical and Health Sciences, Asia University, Taichung City, Taiwan

**Keywords:** acute myocardial infarction, standard modifiable cardiovascular disease risk factors, STEMI, NSTEMI, mortality, SMuRF

## Abstract

**Background:**

Acute myocardial infarction (AMI) cases have decreased in part due to the advent of targeted therapies for standard modifiable cardiovascular disease risk factors (SMuRF). Recent studies have reported that ST-elevation myocardial infarction (STEMI) patients without SMuRF (termed “SMuRF-less”) may be increasing in prevalence and have worse outcomes than “SMuRF-positive” patients. As these studies have been limited to STEMI and comprised mainly Caucasian cohorts, we investigated the changes in the prevalence and mortality of both SMuRF-less STEMI and non-STEMI (NSTEMI) patients in a multiethnic Asian population.

**Methods:**

We evaluated 23,922 STEMI and 62,631 NSTEMI patients from a national multiethnic registry. Short-term cardiovascular and all-cause mortalities in SMuRF-less patients were compared to SMuRF-positive patients.

**Results:**

The proportions of SMuRF-less STEMI but not of NSTEMI have increased over the years. In hospitals, all-cause and cardiovascular mortality and 1-year cardiovascular mortality were significantly higher in SMuRF-less STEMI after adjustment for age, creatinine, and hemoglobin. However, this difference did not remain after adjusting for anterior infarction, cardiopulmonary resuscitation (CPR), and Killip class. There were no differences in mortality in SMuRF-less NSTEMI. In contrast to Chinese and Malay patients, SMuRF-less patients of South Asian descent had a two-fold higher risk of in-hospital all-cause mortality even after adjusting for features of increased disease severity.

**Conclusion:**

SMuRF-less patients had an increased risk of mortality with STEMI, suggesting that there may be unidentified nonstandard risk factors predisposing SMuRF-less patients to a worse prognosis. This group of patients may benefit from more intensive secondary prevention strategies to improve clinical outcomes.

## Introduction

Cardiovascular disease (CVD) is one of the leading causes of death and disability worldwide (1, 2). The incidence of acute myocardial infarction (AMI) has been reported to be about 300 per 100,000 people or greater, depending on the country or ethnicity (3). Cases of hospitalization due to AMI have decreased over the past few decades presumably due to the advent of targeted therapies for specific risk factors. The reduction in AMI morbidity and mortality has been achieved in part through secondary prevention of CVD along with other non-pharmacological interventions and measures, such as lifestyle modification and the adoption of community screening (4, 5). Hypertension, hyperlipidemia, diabetes, obesity, and cigarette smoking are well-recognized standard modifiable cardiovascular risk factors (SMuRF) that are commonly associated with CVD and are therefore used to evaluate the risk of AMI in patients (6).

Interestingly, recent studies have suggested that the number of AMI patients without apparent SMuRF (termed “SMuRF-less”) has increased over the years (7, 8). In an initial retrospective study from a single center in Australia, the prevalence of SMuRF-less ST-elevation myocardial infarction (STEMI) patients increased from 11 to 27% over 8 years (8), a finding that was also observed in a large study cohort from the Australian national registry, where the proportion of SMuRF-less STEMI patients increased from 14 to 23% over a period of 18 years (7). In contrast, a recent study based on the Swedish national registry displayed no evidence of increasing numbers of SMuRF-less STEMI patients, suggesting that the emergence of the SMuRF-less population may be ethnicity- or cohort-dependent and should be validated in other settings (9). Nonetheless, these studies have consistently reported that SMuRF-less STEMI patients have poorer clinical outcomes when compared to SMuRF-positive STEMI patients, such as in-hospital all-cause mortality, suggesting that the care for this population is suboptimal, presumably due to the lack of understanding of the mechanisms contributing to the worse outcomes in this group of patients (7, 9, 10).

Therefore, these studies have raised the awareness of a traditionally neglected group of STEMI patients without any apparent modifiable CVD risk factors. However, the following questions remain unanswered: (1) whether the proportion of SMuRF-less patients for AMI is increasing in different ethnic groups and (2) whether SMuRF-less status is associated with worse mortality outcomes in STEMI and non-STEMI (NSTEMI) patients, compared to the SMuRF-positive patients. As such, to address these issues, we analyzed data from a national population-based multiethnic Asian AMI registry in Singapore.

## Methods

### Data Sources

Data from the Singapore Myocardial Infarction Registry (SMIR) from January 2008 to June 2018 were utilized for this study. The SMIR is a national, government-funded registry, maintained by the National Registry of Diseases Office (NRDO) in Singapore (11). This study received an exemption review (SingHealth CIRB Reference No. 2016/2480) with a waiver for informed consent from patients as the study was based on de-identified data. The study was performed in accordance with the Declaration of Helsinki. The statistician was the only person who had access to the anonymized individual-level data, while the rest of the coauthors had access to the analyzed and aggregated data.

The SMIR database contains demographic, clinical, and outcome data of all AMI patients from the public and private hospitals in Singapore. Notification of AMI by healthcare professionals to the registry is mandated by law (12). The notification sources include medical claims listings, patient discharge summaries, and laboratory results, based on the International Classification of Diseases 9th Revision (ICD-9) Clinical Modification code 410 prior to 2012 and the ICD-10 Australian Modification codes I21 and I22 from 2012 onward. The registry coordinators collected detailed individual-level data from the medical records after ascertaining that the notified cases were, in fact, AMI. The quality of SMIR data was maintained through annual audits for accuracy and inter-rater reliability and review for outliers and illogical data. The SMIR data were subsequently merged with the death data from the Registry of Births and Deaths to obtain mortality outcomes. It is mandatory to report all deaths in Singapore.

### Data Definitions and Exclusion Criteria

The classification of the type of myocardial infarction was based on documentation in the medical records. STEMI was defined by (1) typical chest pain of 20 min, (2) significant ST-segment elevation (0.1 or 0.2 mV on 2 adjacent limb or precordial leads, respectively, or new left bundle-branch block), and (3) confirmed later by a raise in biomarkers. NSTEMI was defined by (1) typical chest pain, (2) new horizontal or downsloping ST-depression (0.05 mV in 2 contiguous leads and/or *T* inversion >0.1 mV in two contiguous leads with prominent R wave or R/S ratio >1), and (3) confirmed by a raise in biomarkers (8). Troponin (T or I) was defined as abnormal if the value was >99th percentile of the reference range for each hospital's laboratory (11). Smoking included ex- and current smokers, regardless of when the ex-smokers stopped smoking and regardless of the type, frequency, and number of cigarettes smoked for current smokers. Data on smoking were self-reported during the admission for AMI. Obesity referred to a body mass index of ≥27.5 kg/m^2^, the cutoff value for Asian populations (13). Data on body mass index was based on the weight measured during the admission for AMI and the latest measured height documented in the medical records. We did not assess abdominal obesity as waist circumference data were not captured by the registry. Medical histories of hypertension, diabetes, and hyperlipidemia were based on past diagnoses and treatments given. Newly diagnosed hypertension was based on systolic blood pressure >130 bpm or diastolic blood pressure >85 bpm in the admission for AMI. Newly diagnosed diabetes was based on fasting blood glucose ≥7.0 mmol/L or random glucose ≥11.1 mmol/L in the admission for AMI. Newly diagnosed hyperlipidemia was based on total cholesterol >6.2 mmol/L, low-density lipoprotein (LDL) cholesterol >4.1 mmol/L, or triglyceride >1.7 mmol/L in the admission for AMI. We used two different definitions of SMuRF status as follows: (1) hypertension, diabetes, hyperlipidemia, smoking, and obesity; and (2) the same SMuRF but excluding obesity to reflect previously published analyses more closely in other ethnic groups. Some preceding studies did not include obesity as one of the SMuRF, but obesity was included in this study as it is one of the important risk factors in this study population (14).

In this study, only AMI patients who underwent percutaneous coronary intervention (PCI) were included. AMI patients who were treated medically without PCI were excluded as this population was heterogeneous and the majority of the patients had type 2 MI. Patients who had at least one risk factor with unknown status due to the nonavailability of data were excluded. Patients with a known history of AMI, PCI, or coronary artery bypass graft surgery were excluded. For patients with recurrent AMI, their first AMI was included, but subsequent AMIs were excluded.

The mortality outcomes in this study were in-hospital all-cause mortality, in-hospital cardiovascular mortality, 1-year (from the onset of AMI) all-cause mortality, and 1-year cardiovascular mortality. Cardiovascular mortality included the following ICD-9 and ICD-10 codes for the identification of cardiovascular death: AMI (410 for ICD-9 and I21 for ICD-10), unstable angina (411.1 and 411.8 for ICD-9 and I20.0 for ICD-10), heart failure (428.0, 428.1, 428.9, and 402.9 for ICD-9 and I50 for ICD-10), acute ischemic stroke (433, 434, and 436 for ICD-9 and I63 for ICD-10), and transient ischemic attack (435 for ICD-9 and G45.9 for ICD-10).

### Statistical Analysis

Categorical variables were expressed as the frequency with percentages, while continuous variables were expressed as median with interquartile range. The chi-square test was used to compare the categorical variables between the SMuRF-less and SMuRF-positive patients, while the Wilcoxon rank-sum test was used to compare the continuous variables between the two groups. Missing data were excluded from analyses through case deletion without imputation in order to maintain data in its original form.

Cox regression was performed to determine the hazard ratios (HR) of mortality for SMuRF-less patients compared to SMuRF-positive patients. Age, creatinine on admission, and hemoglobin on admission were included in the multivariable Cox regression model 1. Age, Killip class on admission, CPR on admission, creatinine on admission, and hemoglobin on admission were included in the multivariable Cox regression model 2. Additionally, the type of myocardial infarction and anterior infarct was included in the models for all AMI and STEMI patients, respectively. These variables were selected based on stepwise backward elimination, starting with all variables captured by the SMIR. All analyses were stratified into STEMI and NSTEMI and replicated for the SMuRF that included and excluded obesity. Subgroup analyses by gender, ethnicity, and age group were undertaken to see if the effect of SMuRF differed across the various subsets of patients.

Linear regression with the logarithmic annual proportion of SMuRF-less patients as outcome and calendar year as a covariate was performed to analyze the trend of SMuRF-less prevalence over the years.

Statistical analysis was performed using Stata (StataCorp. 2013. *Stata Statistical Software: Release 13*. College Station, TX: StataCorp LP). All statistical tests were 2-tailed, and results were deemed to be statistically significant if *p* < 0.05.

## Results

### Study Population

**Figure 1:**
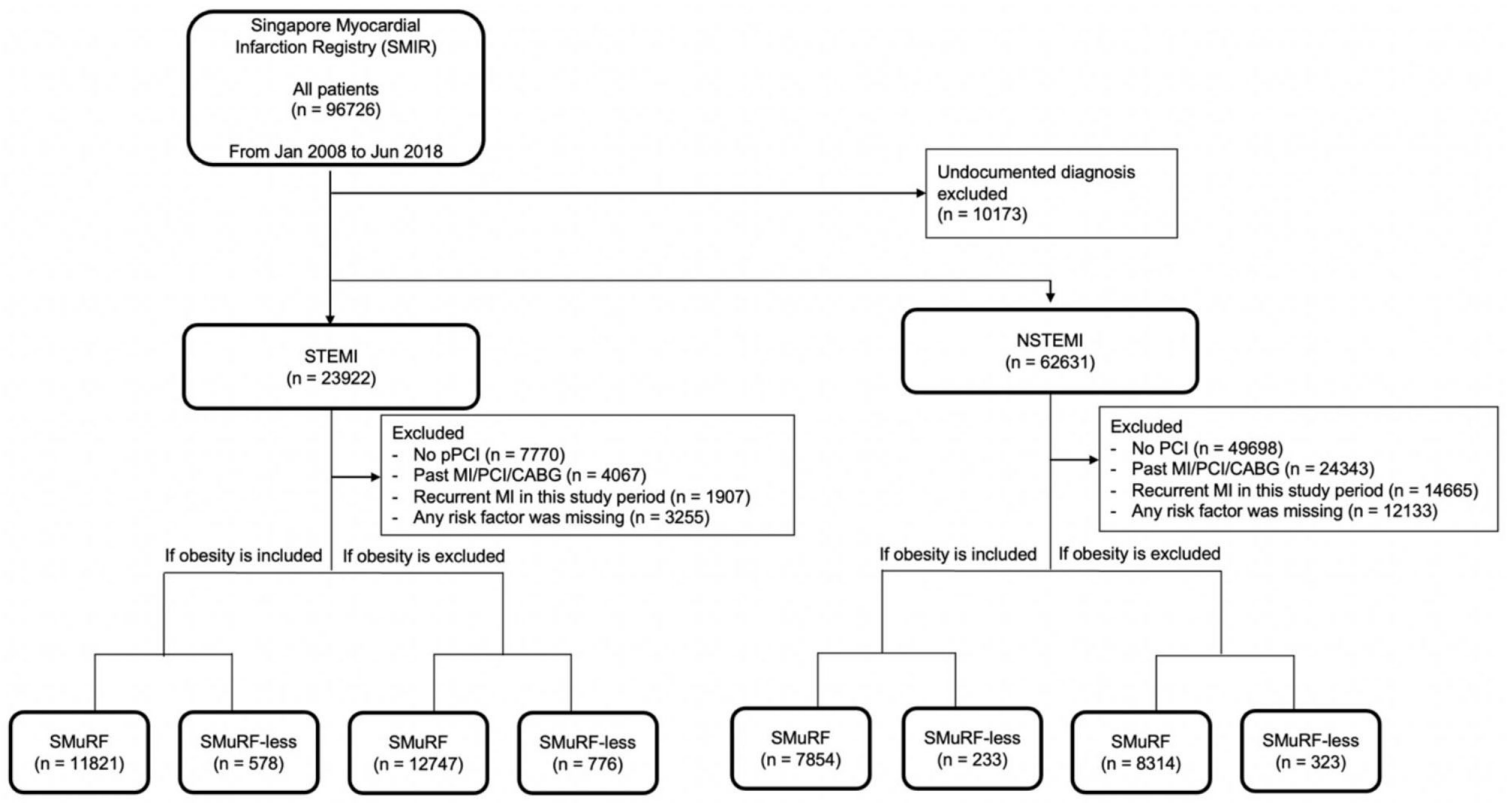
Flowchart of data inclusion. The following criteria for exclusion were applied in this selection: (1) undocumented diagnosis, (2) no percutaneous intervention (PCI) performed, (3) past MI/PCI/coronary arterial bypass surgery (CABG), (4) recurrent MI in this study period, and (5) any risk factor missing. For both STEMI and NSTEMI, obesity was included or excluded to evaluate the impact of obesity as a risk factor in AMI.

When obesity was included as a SMuRF, 11,821 (95.3%) STEMI patients had at least one risk factor, while 578 (4.7%) STEMI patients did not have any of the five risk factors (Figure 1), only 4.7% of the STEMI population were SMuRF-less. Similarly, 7,854 (97.1%) and 233 (2.9%) NSTEMI patients were SMuRF-positive and SMuRF-less, respectively. For STEMI, smoking was the most common risk factor, followed by hyperlipidemia, hypertension, diabetes, and obesity (Table 1), whereas for NSTEMI, hyperlipidemia was the most common risk factor, followed by hypertension, smoking, diabetes, and obesity. Compared to SMuRF-less patients, SMuRF-positive STEMI patients tended to be younger, while SMuRF-positive NSTEMI patients tended to be older. The proportions of men and non-Chinese were higher in both STEMI and NSTEMI SMuRF-positive patients. SMuRF-positive STEMI patients had a lower proportion of Killip class 4 on admission, while SMuRF-positive NSTEMI patients had a lower proportion of Killip class 1 on admission. SMuRF was associated with increased serum creatinine and a less favorable lipid profile in both STEMI and NSTEMI, and increased hemoglobin in STEMI. The proportions of SMuRF-positive STEMI patients who received aspirin, angiotensin-receptor converting enzyme inhibitor/angiotensin receptor blocker (ACEI/ARB), a lipid-lowering drug, and P2Y12 inhibitor during their hospitalization were higher than SMuRF-less. Similarly, the proportions of SMuRF-positive NSTEMI patients who received beta-blockers and ACEI/ARB during hospitalization were higher than SMuRF-less.

**Table 1:** Baseline characteristics of STEMI and NSTEMI patients with respect to SMuRF (including obesity) (*n* = 20,476).

	**Overall**			**STEMI**			**NSTEMI**		
	**SMuRF-less** ***n =* 811**	**≥1 SMuRF** ***n =* 19,675**	***P*** **-value**	**SMuRF-less** ***n =* 578**	**≥1 SMuRF** ***n =* 11,821**	***P*** **-value**	**SMuRF-less** ***n =* 233**	**≥1 SMuRF** ***n =* 7,854**	***P*** **-value**
**Demographics**									
Age in years, median (IQR)	58.9 (51.9–67.3)	58.2 (51.2–66.6)	0.042	59.9 (52.2–67.8)	57.7 (50.8–65.7)	<0.001	58.0 (51.6–66.0)	59.2 (51.7–67.9)	0.242
Male, *n* (%)	607 (74.9)	16,238 (82.5)	<0.001	436 (75.4)	10,067 (85.2)	<0.001	171 (73.4)	6,171 (78.6)	0.058
**Race**, ***n*** **(%)**									
Chinese	614 (75.7)	12,487 (63.5)	<0.001	432 (74.7)	7,432 (62.9)	<0.001	182 (78.1)	5,055 (64.4)	<0.001
Malay	59 (7.3)	3,865 (19.6)		38 (6.6)	2,409 (20.4)		21 (9.0)	1,456 (18.5)	
South Asian	119 (14.7)	2,989 (15.2)		92 (15.9)	1,788 (15.1)		27 (11.6)	1,201 (15.3)	
Others	19 (2.3)	334 (1.7)		16 (2.8)	192 (1.6)		3 (1.3)	142 (1.8)	
**Risk factors**									
Hypertension, *n* (%)	0	11,348 (57.7)		0	6,282 (53.1)		0	5,066 (64.5)	
Diabetes, *n* (%)	0	7,341 (37.3)		0	4,242 (35.9)		0	3,099 (39.5)	
Hyperlipidemia, *n* (%)	0	13,035 (66.3)		0	7,381 (62.4)		0	5,654 (72.0)	
**Smoking status**, ***n*** **(%)**									
Current	0	9,165 (46.6)		0	6,052 (51.2)		0	3,113 (39.6)	
Former	0	2,817 (14.3)		0	1,549 (13.1)		0	1,268 (16.1)	
Never	811 (100)	7,693 (39.1)		578 (100)	4,220 (35.7)		233 (100)	3,473 (44.2)	
Obese, *n* (%)	0	5,356 (27.2)		0	3,025 (25.6)		0	2,331 (29.7)	
Body mass index in kg/m^2^, median (IQR)	23.3 (21.5–24.9)	24.9 (22.6–27.8)		23.1 (21.5–24.9)	24.8 (22.5–27.6)		23.4 (21.5–25.0)	25.1 (22.8–28.1)	
**Total number of SMuRFs**, ***n*** **(%)**									
0	811 (100)	0		578 (100)	0		233 (100)	0	
1	0	4,105 (20.9)		0	2,764 (23.4)		0	1,341 (17.1)	
2	0	6,237 (31.7)		0	3,823 (32.3)		0	2,414 (30.7)	
3	0	5,570 (28.3)		0	3,204 (27.1)		0	2,366 (30.1)	
4	0	3,042 (15.5)		0	1,641 (13.9)		0	1,401 (17.8)	
5	0	721 (3.7)		0	389 (3.3)		0	332 (4.2)	
Total cholesterol in mmol/L, median (IQR)	4.9 (4.3–5.3)	5.2 (4.3–6.0)	<0.001	4.8 (4.3–5.3)	5.1 (4.4–6.0)	<0.001	5.0 (4.6–5.5)	5.2 (4.3–6.1)	0.001
HDL cholesterol in mmol/L, median (IQR)	1.1 (0.9–1.4)	1.0 (0.9–1.2)	<0.001	1.1 (1.0–1.4)	1.0 (0.9–1.2)	<0.001	1.1 (0.9–1.3)	1.0 (0.9–1.2)	<0.001
Triglyceride in mmol/L, median (IQR)	1.1 (0.8–1.6)	1.5 (1.0–2.1)	<0.001	1.1 (0.8–1.5)	1.4 (1.0–2.0)	<0.001	1.3 (0.9–1.9)	1.6 (1.1–2.3)	<0.001
LDL cholesterol in mmol/L, median (IQR)	3.2 (2.7–3.6)	3.4 (2.6–4.2)	<0.001	3.1 (2.7–3.6)	3.4 (2.7–4.2)	<0.001	3.3 (2.9–3.7)	3.4 (2.6–4.2)	0.039
**Killip class on admission**, ***n*** **(%)**									
I	706 (87.2)	16,862 (85.7)	<0.001	487 (84.4)	10,036 (84.9)	<0.001	219 (94.0)	6,826 (86.9)	0.003
II	20 (2.5)	1,037 (5.3)		14 (2.4)	491 (4.1)		6 (2.6)	546 (6.9)	
III	14 (1.7)	795 (4.0)		10 (1.7)	399 (3.4)		4 (1.7)	396 (5.0)	
IV	70 (8.6)	975 (5.0)		66 (11.4)	892 (7.5)		4 (1.7)	83 (1.1)	
CPR on admission, *n* (%)	50 (6.2)	472 (2.4)	<0.001	47 (8.1)	432 (3.7)	<0.001	3 (1.3)	40 (0.5)	0.107
Serum creatinine in μmol on admission, median (IQR)	84 (71–98)	87 (74–104)	<0.001	87 (73–101)	89 (76–106)	<0.001	78 (67–90)	84 (72–101)	<0.001
Hemoglobin in g/dL on admission, median (IQR)	14.3 (13.1–15.2)	14.5 (13.3–15.6)	<0.001	14.4 (13.1–15.3)	14.7 (13.5–15.7)	<0.001	14.0 (13.0–15.1)	14.2 (13.0–15.3)	0.147
Abnormal troponin, *n* (%)	754 (93.3)	18,119 (92.3)	0.292	548 (95.3)	11,375 (96.5)	0.120	206 (88.4)	6,744 (86.0)	0.290
Anterior infarct, *n* (%)	NA	NA	NA	331 (57.3)	5,855 (49.5)	<0.001	NA	NA	NA
**Treatment and condition during hospitalization**									
Aspirin, *n* (%)	776 (95.7)	19,145 (97.3)	0.006	544 (94.1)	11,453 (96.9)	<0.001	232 (99.6)	7,692 (97.9)	0.080
Beta blocker, *n* (%)	677 (83.5)	17,082 (86.8)	0.006	482 (83.4)	10,169 (86.0)	0.076	195 (83.7)	6,913 (88.0)	0.046
ACEI/ARB, *n* (%)	522 (64.4)	14,627 (74.3)	<0.001	382 (66.1)	8,762 (74.1)	<0.001	140 (60.1)	5,865 (74.7)	<0.001
Lipid lowering drug, *n* (%)	772 (95.2)	19,236 (97.8)	<0.001	545 (94.3)	11,490 (97.2)	<0.001	227 (97.4)	7,746 (98.6)	0.126
P2Y12 inhibitor, *n* (%)	786 (96.9)	19,405 (98.6)	<0.001	553 (95.7)	11,599 (98.1)	<0.001	233 (100)	7,806 (99.4)	0.231
Lowest LVEF in % during hospitalization, mean (SD)	46.9 (12.8)	47.0 (12.3)	0.840	44.8 (12.5)	44.7 (11.9)	0.729	52.6 (11.8)	50.9 (12.2)	0.033

*ACEI/ARB, angiotensin-receptor converting enzyme inhibitor/angiotensin receptor blocker; CPR, cardiopulmonary resuscitation; IQR, interquartile range; LVEF, left ventricular ejection fraction; NA, not applicable; NSTEMI, non-ST-segment elevation myocardial infarction; SD, standard deviation; SMuRF, standard modifiable cardiovascular risk factors; STEMI, ST-segment elevation myocardial infarction*.

When obesity was excluded from the SMuRF, 12,747 (94.3%) STEMI patients had at least one risk factor, while 776 (5.7%) STEMI patients did not have any of the four risk factors (Figure 1). Similarly, 8,314 (96.3%) and 323 (3.7%) patients were SMuRF and SMuRF-less, respectively. The profile of risk factors was similar to the results where obesity was included as a SMuRF (Table 2).

**Table 2:** Baseline characteristics of STEMI and NSTEMI patients with respect to SMuRF (excluding obesity) (*n* = 22,160).

	**Overall**			**STEMI**			**NSTEMI**		
	**SMuRF-less** ***n =* 1099**	**≥1 SMuRF** ***n =* 21061**	***p*** **-value**	**SMuRF-less** ***n =* 776**	**≥1 SMuRF** ***n =* 12747**	***p*** **-value**	**SMuRF-less** ***n =* 323**	**≥1 SMuRF** ***n =* 8314**	***p*** **-value**
**Demographics**									
Age in years, median (IQR)	58.3 (51.4–66.4)	58.3 (51.2–66.8)	0.977	58.7 (51.6–67.1)	57.8 (50.8–65.9)	0.017	57.3 (50.8–64.4)	59.3 (51.9–68.2)	0.003
Male, *n* (%)	830 (75.5)	17,378 (82.5)	<0.001	592 (76.3)	10,855 (85.2)	<0.001	238 (73.7)	6,523 (78.5)	0.041
**Race**, ***n*** **(%)**									
Chinese	797 (72.5)	13,401 (63.6)	<0.001	554 (71.4)	8,038 (63.1)	<0.001	243 (75.2)	5,363 (64.5)	<0.001
Malay	98 (8.9)	4,159 (19.7)		68 (8.8)	2,601 (20.4)		30 (9.3)	1,558 (18.7)	
South Asian	180 (16.4)	3,150 (15.0)		135 (17.4)	1,904 (14.9)		45 (13.9)	1,246 (15.0)	
Others	24 (2.2)	351 (1.7)		19 (2.4)	204 (1.6)		5 (1.5)	147 (1.8)	
**Risk factors**									
Hypertension, *n* (%)	0	12,233 (58.1)		0	6,831 (53.6)		0	5,402 (65.0)	
Diabetes, *n* (%)	0	7,876 (37.4)		0	4,606 (36.1)		0	3,270 (39.3)	
Hyperlipidemia, *n* (%)	0	14,030 (66.6)		0	8,025 (63.0)		0	6,005 (72.2)	
**Smoking status**, ***n*** **(%)**									
Current	0	9,945 (47.2)		0	6,629 (52.0)		0	3,316 (39.9)	
Former	0	3,043 (14.5)		0	1,680 (13.2)		0	1,363 (16.4)	
Never	1,099 (100)	8,073 (38.3)		776 (100)	4,438 (34.8)		323 (100)	3,635 (43.7)	
**Total number of SMuRFs**, ***n*** **(%)**									
0	1,099 (100)	0		776 (100)	0		323 (100)	0	
1	0	5,335 (25.3)		0	3,536 (27.7)		0	1,799 (21.6)	
2	0	7,493 (35.6)		0	4,584 (36.0)		0	2,909 (35.0)	
3	0	6,126 (29.1)		0	3,441 (27.0)		0	2,685 (32.3)	
4	0	2,107 (10.0)		0	1,186 (9.3)		0	921 (11.1)	
Obese, *n* (%)	185 (18.6)	5,171 (26.5)	<0.001	127 (18.0)	2,898 (24.8)	<0.001	58 (19.9)	2,273 (29.2)	0.001
Body mass index in kg/m^2^, median (IQR)	24.0 (21.9–26.4)	24.9 (22.6–27.7)	<0.001	24.0 (21.9–26.3)	24.7 (22.4–27.5)	<0.001	24.0 (21.8–26.7)	25.1 (22.8–28.1)	<0.001
Total cholesterol in mmol/L, median (IQR)	4.9 (4.4–5.4)	5.2 (4.4–6.0)	<0.001	4.8 (4.3–5.3)	5.2 (4.4–6.0)	<0.001	5.1 (4.6–5.5)	5.2 (4.3–6.1)	0.001
HDL cholesterol in mmol/L, median (IQR)	1.1 (0.9–1.3)	1.0 (0.9–1.2)	<0.001	1.1 (0.9–1.3)	1.0 (0.9–1.2)	<0.001	1.1 (0.9–1.3)	1.0 (0.9–1.2)	0.001
Triglyceride in mmol/L, median (IQR)	1.2 (0.9–1.7)	1.5 (1.0–2.1)	<0.001	1.1 (0.8–1.6)	1.4 (1.0–2.0)	<0.001	1.4 (1.0–2.0)	1.6 (1.1–2.3)	<0.001
LDL cholesterol in mmol/L, median (IQR)	3.2 (2.8–3.6)	3.4 (2.7–4.2)	<0.001	3.2 (2.7–3.6)	3.4 (2.7–4.2)	<0.001	3.3 (2.9–3.7)	3.4 (2.6–4.2)	0.021
**Killip class on admission**, ***n*** **(%)**									
I	958 (87.2)	17,981 (85.4)	<0.001	653 (84.3)	10,761 (84.4)	<0.001	305 (94.4)	7,220 (86.9)	<0.001
II	25 (2.3)	1,125 (5.3)		17 (2.2)	536 (4.2)		8 (2.5)	589 (7.1)	
III	22 (2.0)	859 (4.1)		17 (2.2)	446 (3.5)		5 (1.5)	413 (5.0)	
IV	93 (8.5)	1,090 (5.2)		88 (11.3)	1,001 (7.8)		5 (1.5)	89 (1.1)	
CPR on admission, *n* (%)	67 (6.1)	532 (2.5)	<0.001	63 (8.1)	488 (3.8)	<0.001	4 (1.2)	44 (0.5)	0.093
Serum creatinine in μmol on admission, median (IQR)	85 (71–100)	87 (74–105)	<0.001	88 (74–103)	89 (76–107)	0.002	78 (66–91)	84 (72–101)	<0.001
Hemoglobin in g/dL on admission, median (IQR)	14.3 (13.2–15.3)	14.5 (13.3–15.6)	<0.001	14.5 (13.2–15.4)	14.7 (13.5–15.7)	<0.001	14.0 (13.0–15.1)	14.2 (13.0–15.3)	0.192
Abnormal troponin, *n* (%)	1,018 (92.9)	19,371 (92.2)	0.409	736 (95.2)	12,233 (96.3)	0.132	282 (87.3)	7,138 (86.0)	0.493
Anterior infarct, *n* (%)	NA	NA	NA	439 (56.6)	6,328 (49.6)	<0.001	NA	NA	NA
**Treatment during hospitalization**									
Aspirin, *n* (%)	1,039 (94.5)	20,371 (96.7)	<0.001	720 (92.8)	12,236 (96.0)	<0.001	319 (98.8)	8,135 (97.9)	0.263
Beta blocker, *n* (%)	919 (83.6)	18,071 (85.8)	0.044	639 (82.4)	10,786 (84.6)	0.090	280 (86.7)	7,285 (87.6)	0.617
ACEI/ARB, *n* (%)	704 (64.1)	15,457 (73.4)	<0.001	509 (65.6)	9,294 (72.9)	<0.001	195 (60.4)	6,163 (74.1)	<0.001
Lipid lowering drug, *n* (%)	1,033 (94.0)	20,438 (97.0)	<0.001	719 (92.7)	12,257 (96.2)	<0.001	314 (97.2)	8,181 (98.4)	0.100
P2Y12 inhibitor, *n* (%)	1,054 (95.9)	20,651 (98.1)	<0.001	733 (94.5)	12,390 (97.2)	<0.001	321 (99.4)	8,261 (99.4)	0.968
Lowest LVEF in % during hospitalization, mean (SD)	47.2 (12.5)	46.9 (12.4)	0.618	45.1 (12.3)	44.6 (11.9)	0.253	52.8 (11.3)	50.9 (12.2)	0.016

*ACEI/ARB, angiotensin-receptor converting enzyme inhibitor/angiotensin receptor blocker; CPR, cardiopulmonary resuscitation; IQR, interquartile range; LVEF, left ventricular ejection fraction; NA, not applicable; NSTEMI, non-ST-segment elevation myocardial infarction; SD, standard deviation; SMuRF, standard modifiable cardiovascular risk factors; STEMI, ST-segment elevation myocardial infarction*.

Regardless of whether obesity was included as a SMuRF, SMuRF-less AMI patients were found to have a higher prevalence of increased disease severity compared to SMuRF-positive AMI patients, where a higher proportion of SMuRF-less patients had a higher Killip class, anterior infarction, and received CPR (Tables 1, 2).

**Figure 2:**
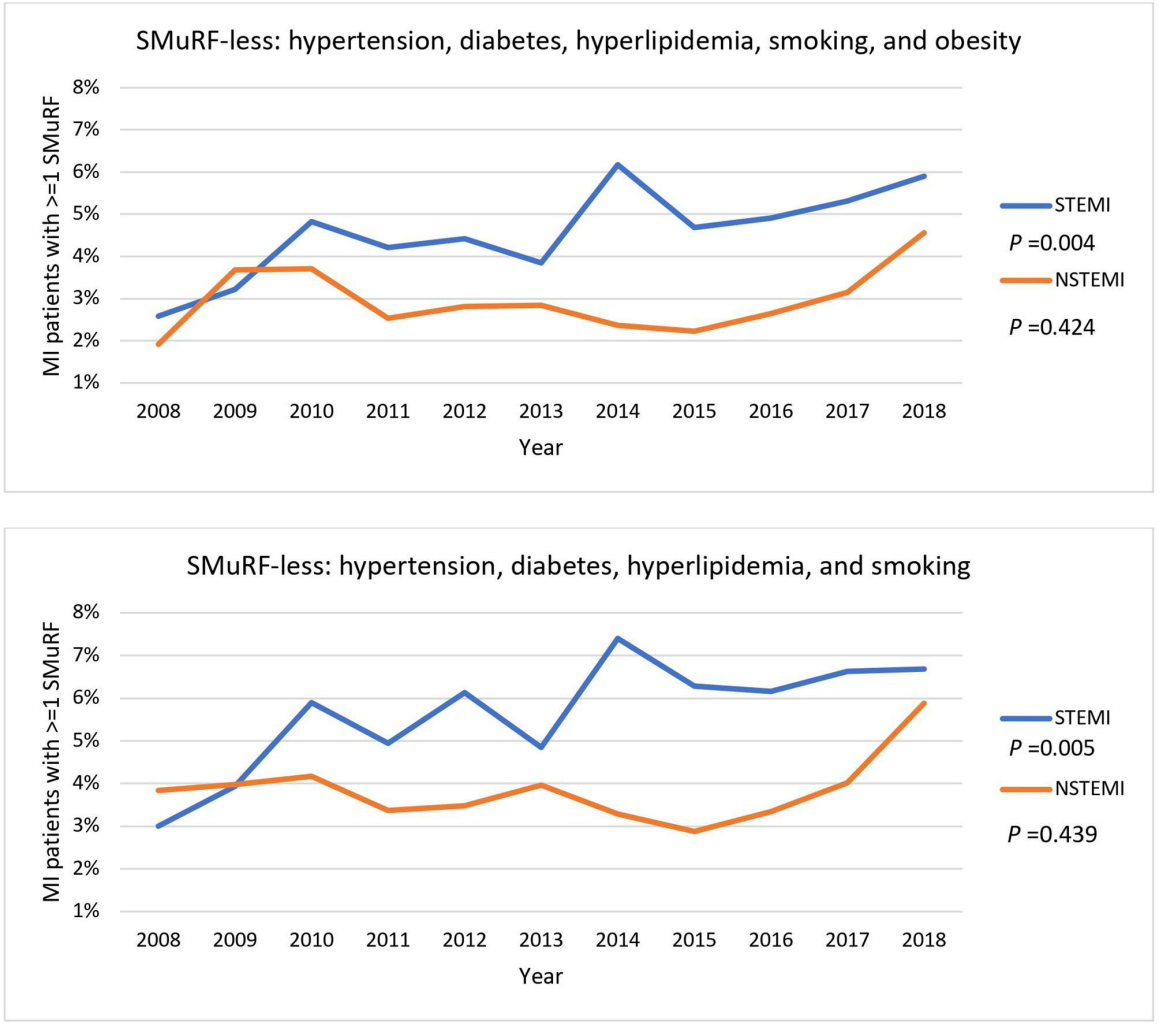
General trends of STEMI and NSTEMI patients without SMuRF from 2008 to 2018. Percentages of STEMI and NSTEMI patients with one or more of SMuRF were traced from 2008 to 2018. Obesity was included as one of the SMuRF (top) or excluded (bottom). The *P-*values represent the statistical significance for the overall trend of STEMI and NSTEMI.

There was a general upward trend in the proportion of SMuRF-less STEMI patients over the past 10 years regardless of including obesity as one of the SMuRF (Figure 2). However, the magnitude of this increase was modest with a 3.3% and 3.7% increase over the period, respectively. The proportion of SMuRF-less NSTEMI patients only started rising from 2015 onward.

### Mortality Outcomes

With the inclusion of obesity as one of the SMuRF, the unadjusted HR for in-hospital all-cause mortality was 53% higher for SMuRF-less STEMI patients (HR 1.53, 95% CI 1.12–2.10) compared to SMuRF-positive STEMI patients (Table 3). After adjusting for age, creatinine, and hemoglobin, the HR for in-hospital all-cause mortality remained statistically significant for STEMI patients with no SMuRF (HR 1.54, 95% CI 1.12–2.11 for model 1). However, when Killip class, CPR, and anterior infarct were included for adjustment, the HR for in-hospital all-cause mortality was no longer statistically significant for STEMI patients with no SMuRF (HR 1.31, 95% CI 0.95–1.81 for model 2). For in-hospital cardiovascular mortality and 1-year all-cause mortality, a similar pattern was observed among the STEMI patients, where the HRs of the SMuRF-less attenuated substantially after adjustment, though the HRs were not significant. Similarly, the unadjusted HR for 1-year cardiovascular mortality was 54% higher for STEMI patients with no SMuRF (HR 1.54, 95% CI 1.08–2.19). However, with adjustment, the statistical significance was lost (HR 1.39, 95% CI 0.97–1.99 for model 1; HR 1.16, 95% CI 0.80–1.67 for model 2). The nonsignificantly higher mortality hazards for NSTEMI SMuRF-less patients all dropped after Killip class, CPR, and anterior infarct were included in the models.

**Table 3:** Risk of death among SMuRF-less AMI patients compared to those with at least one SMuRF (reference).

	**Including obesity as a SMuRF**			**Excluding obesity as a SMuRF**		
	**Overall** **HR (95% CI)**	**STEMI** **HR (95% CI)**	**NSTEMI** **HR (95% CI)**	**Overall** **HR (95% CI)**	**STEMI** **HR (95% CI)**	**NSTEMI** **HR (95% CI)**
**In-hospital all-cause mortality**						
Unadjusted model	1.72 (1.27–2.32)	1.53 (1.12–2.10)	1.50 (0.55–4.08)	1.63 (1.26–2.10)	1.45 (1.11–1.90)	1.36 (0.56–3.33)
Adjusted model 1	1.51 (1.12–2.04)	1.54 (1.12–2.11)	1.27 (0.46–3.50)	1.45 (1.13–1.88)	1.48 (1.13–1.93)	1.26 (0.51–3.10)
Adjusted model 2	1.30 (0.96–1.76)	1.31 (0.95–1.81)	0.97 (0.35–2.71)	1.22 (0.94–1.59)	1.20 (0.92–1.58)	1.01 (0.40–2.50)
**In-hospital cardiovascular mortality**						
Unadjusted model	1.64 (1.15–2.35)	1.44 (0.99–2.08)	1.12 (0.27–4.62)	1.63 (1.22–2.18)	1.43 (1.06–1.93)	1.18 (0.37–3.75)
Adjusted model 1	1.38 (0.96–1.99)	1.41 (0.97–2.06)	1.03 (0.24–4.50)	1.41 (1.05–1.9)	1.44 (1.06–1.95)	1.18 (0.35–3.94)
Adjusted model 2	1.13 (0.77–1.64)	1.15 (0.78–1.71)	0.71 (0.17–2.94)	1.13 (0.82–1.55)	1.12 (0.80–1.56)	0.84 (0.28–2.54)
**1-year all-cause mortality**						
Unadjusted model	1.25 (0.96–1.62)	1.18 (0.88–1.59)	1.16 (0.63–2.11)	1.16 (0.93–1.46)	1.15 (0.90–1.47)	0.92 (0.53–1.60)
Adjusted model 1	1.09 (0.83–1.42)	1.06 (0.79–1.42)	1.33 (0.73–2.44)	1.09 (0.87–1.36)	1.09 (0.85–1.39)	1.18 (0.68–2.06)
Adjusted model 2	1.04 (0.80–1.36)	0.96 (0.71–1.29)	1.28 (0.70–2.35)	1.03 (0.82–1.30)	0.96 (0.75–1.24)	1.17 (0.67–2.04)
**1-year cardiovascular mortality**						
Unadjusted model	1.61 (1.15–2.26)	1.54 (1.08–2.19)	0.99 (0.31–3.13)	1.50 (1.13–1.99)	1.45 (1.08–1.94)	0.87 (0.32–2.36)
Adjusted model 1	1.35 (0.95–1.90)	1.39 (0.97–1.99)	1.12 (0.35–3.57)	1.35 (1.01–1.8)	1.39 (1.03–1.87)	1.12 (0.41–3.05)
Adjusted model 2	1.18 (0.84–1.67)	1.16 (0.80–1.67)	0.98 (0.31–3.07)	1.15 (0.86–1.54)	1.11 (0.82–1.52)	1.00 (0.39–2.57)

*CI, confidence interval; HR, hazard ratio; NSTEMI, non-ST-elevation myocardial infarction; STEMI, ST-elevation myocardial infarction*.

*The variables included as covariates in adjusted model 1 were age, creatinine, hemoglobin, and MI type (for overall) and in adjusted model 2 were age, Killip class, CPR, creatinine, hemoglobin, MI type (for overall), and anterior infarct (for STEMI)*.

Similarly, with the exclusion of obesity as one of the SMuRF, the unadjusted HR for in-hospital all-cause mortality was 45% higher for STEMI patients without SMuRF (HR 1.45, 95% CI 1.11–1.90) compared to the SMuRF (Table 3). After adjusting for age, creatinine, and hemoglobin, the HR for in-hospital all-cause mortality remained statistically significant for STEMI patients without SMuRF (HR 1.48, 95% CI 1.13–1.93 for model 1). However, when Killip class, CPR, and anterior infarct were included for adjustment, the HR for in-hospital all-cause mortality was no longer statistically significant for STEMI patients without SMuRF (HR 1.20, 95% CI 0.92–1.58 for model 2). Similarly, the unadjusted HRs for in-hospital cardiovascular mortality and 1-year cardiovascular mortality were 43% and 45% higher for STEMI patients without SMuRF (HR 1.43, 95% CI 1.06–1.93 and HR 1.45, 95% CI 1.08–1.94, respectively). These increased HRs remained statistically significant after adjustment only in the adjusted model 1 (in-hospital cardiovascular mortality HR 1.44, 95% CI 1.06–1.95 for model 1; HR 1.12, 95% CI 0.80–1.56 for model 2) and model 2 (1-year cardiovascular mortality HR 1.39, 95% CI 1.03–1.87 for model 1; HR 1.11, 95% CI 0.82–1.52 for model 2). No significant difference in 1-year all-cause mortality was observed between the SMuRF-less and SMuRF-positive STEMI patients. There was also no significant difference in HRs between those with SMuRF and the SMuRF-less for all mortality outcomes among NSTEMI patients.

**Figure 3:**
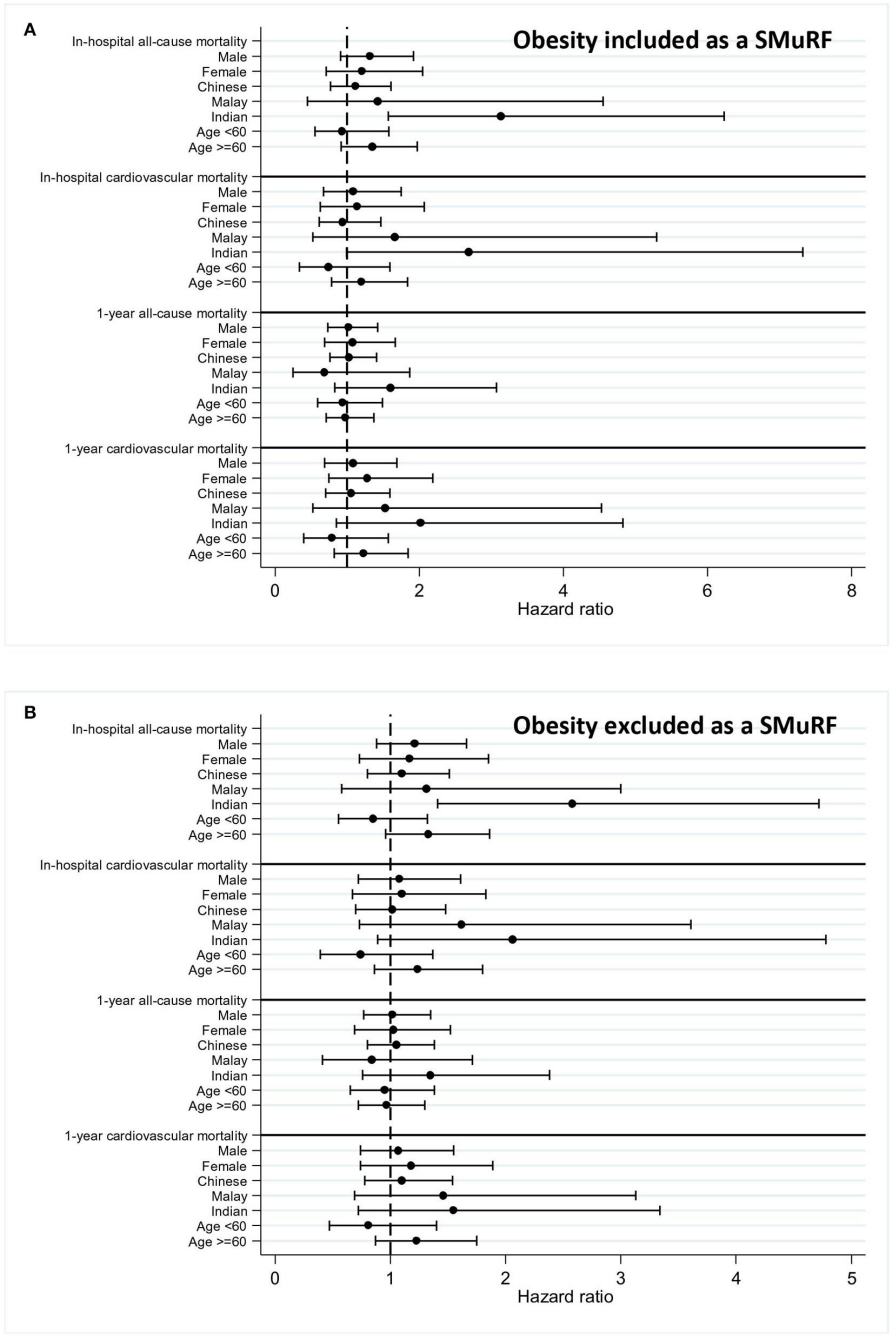
Forest plot for an adjusted hazard ratio of mortality within subgroups. Adjusted hazard ratios of mortalities within subgroups (men, women, Chinese, Malay, South Asian, and age) were calculated and plotted in forest plots for patients with myocardial infarction (MI) with 95% confidence intervals (CI). Obesity was included **(A)** or excluded **(B)** as a SMuRF for the analysis.

Subgroup analysis revealed that there was an increased adjusted HR for in-hospital all-cause mortality within the South Asian SMuRF-less group regardless of the inclusion of obesity as a SMuRF (HR 3.13, 95% CI 1.57–6.23 if obesity was included; HR 2.58, 95% CI 1.41–4.72 if obesity was excluded) (Table 4 and Figure 3).

**Table 4:** Risk of death among SMuRF-less AMI patients compared to those with at least one SMuRF (reference) within subgroups.

	**Including obesity as a SMuRF** **Adjusted HR (95% CI)**	**Excluding obesity as a SMuRF** **Adjusted HR (95% CI)**
**In-hospital all-cause mortality**		
Male	1.32 (0.91–1.92)	1.21 (0.88–1.66)
Female	1.21 (0.71–2.05)	1.17 (0.73–1.85)
Chinese	1.12 (0.77–1.61)	1.10 (0.80–1.51)
Malay	1.42 (0.45–4.55)	1.31 (0.58–3.00)
South Asian	3.13 (1.57–6.23)	2.58 (1.41–4.72)
Young	0.93 (0.55–1.58)	0.85 (0.55–1.32)
Old	1.35 (0.92–1.97)	1.33 (0.96–1.86)
**In-hospital cardiovascular mortality**		
Male	1.08 (0.67–1.75)	1.08 (0.72–1.61)
Female	1.14 (0.63–2.07)	1.10 (0.67–1.83)
Chinese	0.94 (0.61–1.47)	1.02 (0.70–1.48)
Malay	1.66 (0.52–5.30)	1.62 (0.73–3.61)
South Asian	2.69 (0.99–7.33)	2.06 (0.89–4.78)
Young	0.74 (0.34–1.59)	0.74 (0.39–1.37)
Old	1.20 (0.78–1.84)	1.24 (0.86–1.80)
**1-year all-cause mortality**		
Male	1.02 (0.73–1.42)	1.02 (0.77–1.35)
Female	1.07 (0.69–1.67)	1.03 (0.69–1.52)
Chinese	1.03 (0.76–1.41)	1.05 (0.80–1.38)
Malay	0.69 (0.25–1.87)	0.84 (0.41–1.71)
South Asian	1.60 (0.83–3.07)	1.35 (0.76–2.38)
Young	0.94 (0.59–1.49)	0.95 (0.65–1.38)
Old	0.98 (0.71–1.37)	0.97 (0.72–1.30)
**1-year cardiovascular mortality**		
Male	1.08 (0.69–1.69)	1.07 (0.74–1.55)
Female	1.28 (0.75–2.19)	1.18 (0.74–1.89)
Chinese	1.06 (0.70–1.59)	1.10 (0.78–1.54)
Malay	1.53 (0.52–4.53)	1.46 (0.69–3.13)
South Asian	2.02 (0.85–4.83)	1.55 (0.72–3.34)
Young	0.79 (0.40–1.57)	0.81 (0.47–1.40)
Old	1.23 (0.82–1.85)	1.23 (0.87–1.75)

*CI, confidence interval; HR, hazard ratio; MI, myocardial infarction*.

*The following variables were included as covariates in the models used to estimate the adjusted HR: age, Killip class, CPR, creatinine, hemoglobin, MI type (for overall), and anterior infarct (for STEMI)*.

## Discussion

In this national registry-based retrospective study with a multiethnic cohort of both STEMI and NSTEMI patients, we demonstrated that SMuRF-less STEMI patients had a worse in-hospital all-cause mortality and 1-year cardiovascular mortality, regardless of the inclusion of obesity as a risk factor. The proportion of SMuRF-less STEMI patients has increased over time, although this trend was modest. These findings suggest that being SMuRF-less is associated with an increased risk of mortality in STEMI but not NSTEMI patients when compared to SMuRF-positive.

One of the first studies reporting an association between the SMuRF and prognosis of AMI patients was published in 2011 by Canto et al., who showed that the number of CVD risk factors was inversely correlated with in-hospital all-cause mortality in AMI patients in the US cohort (15). Given the pathogenesis of AMI and the roles of these risk factors for the formation of coronary atherosclerosis, this result appeared counterintuitive and was controversial at the time (16–19). Subsequently, the clinical importance and the awareness of SMuRF-less patients were highlighted by two retrospective studies based on an Australian cohort, which reported that the proportion of SMuRF-less among AMI patients had substantially increased over time and was associated with a worse prognosis (7–9). The authors proposed that these studies provided indirect evidence for the existence of other clinically critical but unidentified risk factors in this patient group. Our study, in part, supports this hypothesis. In our multiethnic southeast Asian study, we observed worse mortality outcomes in SMuRF-less compared to SMuRF-positive STEMI patients after adjustment for baseline characteristics (adjusted model 1). However, this finding was no longer significant after adjusting for the significantly higher rates of anterior infarction, cardiopulmonary resuscitation (CPR), and Killip class observed in SMuRF-less (adjusted model 2). These results suggest that although being SMuRF-less is not an independent predictor of clinical outcomes, SMuRF-less patients do have worse mortality outcomes following STEMI. In addition to this finding, our subgroup analysis revealed that the South Asian subgroup had a higher risk of in-hospital death even after adjusting for the significantly higher rates of anterior infarction, CPR, and Killip class observed in SMuRF-less, suggesting the presence of so far unidentified CVD risk factors that can be determinants of worse outcomes in SMuRF-less, such as lipoprotein A (20–22). In support of this, SMuRF-less AMI patients had more favorable lipid profiles (lower LDL and triglyceride levels and higher high-density lipoprotein (HDL) levels) compared to SMuRF-positive patients supporting the presence of unknown CVD risk factors in SMuRF-less patients. Interestingly, although being SMuRF-less was associated with worse mortality outcomes in STEMI, there was no difference with NSTEMI, the reasons for which are not clear but may relate to the differing pathophysiology and risk factor profiles of STEMI vs. NSTEMI.

As explained above, unknown CVD risk factors, such as lipoprotein A, could be a cause of the worse clinical outcomes in SMuRF-less STEMI patients. Another alternative explanation for the worse prognosis is the involvement of the genetic factors predisposing the patients to worse outcomes. In addition to the genetic predisposition or unidentified risk factors, there could be other alternative explanations for the worse prognosis in SMuRF-less STEMI patients. As shown in Table 2, SMuRF-less patients were less likely to receive evidence-based treatment as compared to SMuRF patients. This undertreatment may have contributed to the worse mortalities noted in previous studies (7–9). Furthermore, the presence of coronary collaterals in patients with silent myocardial ischemia, which is often observed in patients with CVD risk factors, could lead to lower mortality in SMuRF patients (23, 24).

Obesity is associated with the incidence of AMI (25–30) and is associated with the premature occurrence of AMI in young patients (31–33). Despite the association between obesity and the incidence of AMI, whether obesity increases the risk of mortalities following AMI events has been controversial. Obesity significantly decreased the risk of mortality outcomes after AMI, and this phenomenon has been termed the “obesity paradox in AMI” (34–40). However, other follow-up studies have demonstrated no difference in mortality outcomes between obese and nonobese patients after STEMI (41, 42) and AMI (33) or even worse prognosis in obese patients following AMI (43). Presumably, due to this controversy, obesity has been excluded as a risk factor in previous SMuRF studies. Our study included two independent datasets with and without obesity as a SMuRF to assess whether the SMuRF-less status performs better as a predictor of clinical outcomes. Regardless of the inclusion of obesity as a risk factor, being SMuRF-less was associated with worse mortality outcomes, although being SMuRF-less was not an independent predictor of mortality.

One potential critique for studies examining the role of SMuRF on outcomes is that accurate identification of risk factors is challenging as this is often based on (1) the patient's self-reported history, (2) medical records, or (3) current medications. To minimize the risk of under-identifying SMuRF, we included not only the three criteria mentioned above but also risk factors confirmed through laboratory tests. For this reason, our study further extends knowledge in the field by strictly defining the SMuRF based on medical records and laboratory values during hospitalization. Moreover, only never smokers were included as nonsmokers, as compared to previous analyses where ex-smokers were considered nonsmokers due to the persistent negative impact of smoking on cardiovascular mortalities even after cessation (7–9, 44–46). Interestingly, although the proportions of SMuRF-less were 25% (8), 19% (7), and 15% (9) for STEMI patients in the previous studies, our study revealed <6% for STEMI and NSTEMI patients without SMuRF, which may, in part, be explained by the detection of SMuRF in our study. An alternative explanation could be that the CVD risk factor burden is greater in our multiethnic AMI population when compared to the Australian and Swedish AMI cohorts.

Currently, it is unclear whether the proportion of AMI patients without any standard CVD risk factors is changing over time worldwide. The only studies that have reported this increasing trend are two investigations in Australia; the proportion of SMuRF-less patients increased from 11% in 2006 to 27% in 2014 in one study and from 14% in 1999 to 23% in 2017 in another study (7, 8). These findings may be clinically important as they may suggest the increasing importance of a traditionally misidentified group with unknown CVD risk factors. However, this trend was not reported in a collaborative follow-up study performed by the original authors using the Swedish national registry, suggesting that this increasing trend in the proportion of the SMuRF-less patients among AMI patients might have been ethnicity-specific (9). This hypothesis is strengthened by the following evidence: in the Japan AMI registry, the proportion of AMI patients with the traditional CVD risk factors, such as hypertension, diabetes mellitus, and hypercholesterolemia, has increased over time (47). Similarly, the mean number of SMuRF increased from 1.76-fold in 1997 to 2.26-fold in 2017 among male AMI patients and from 1.83-fold to 2.24-fold among female AMI patients in the Swiss National AMI Registry (48). Moreover, given the fact that the profile of the SMuRF was significantly different among the aforementioned studies, careful interpretation and application of these findings are warranted (49–54). In our multiethnic AMI cohort, we observed a modest increase in the prevalence of SMuRF-less STEMI patients but no difference with NSTEMI patients.

### Strengths and Limitations

Our study significantly contributes to the field for the following reasons. It is controversial whether the proportion of the SMuRF-less STEMI patients has been increasing as previous studies reported conflicting results. Using a national multiethnic AMI registry, we demonstrated that the incidence of SMuRF-less STEMI has been increasing in this study. Prior studies have focused only on clinical outcomes in SMuRF-less STEMI patients, whereas in our study we included both STEMI and NSTEMI patients. To the best of our knowledge, this is the first time that it has been reported that short-term mortalities were higher in SMuRF-less STEMI patients but not in NSTEMI patients. Taken together, these data suggest that STEMI SMuRF-less patients may require more aggressive medical interventions and attention and the absence of SMuRF should not lead to undertreatment in this group of patients. Additionally, the strength of this study is that the registry of AMI patients ensures near-complete case coverage at the national level with its mandatory notification. Due to the nature of the database and study design, there was minimal risk of selection bias. Furthermore, our study strictly defined patients' risk factors using not only the patient's self-reported history, previous medical records, and current medications but also the results obtained during hospitalization for AMI. Nevertheless, we acknowledge several limitations of this study. Only AMI patients who underwent PCI were included in this study as they were a homogenous group of patients. Therefore, the results of our study are only applicable to patients who received PCI but not other patient populations, such as those who did not receive revascularization (but received medical therapy alone or thrombolysis). While the subgroup analysis revealed that there was an increased adjusted HR for in-hospital all-cause mortality within the South Asian SMuRF-less group, the low prevalence of SMURF-less and low rate of mortality among the patients might lead to statistical bias in the risk estimates. We attempted to keep the multivariable models parsimonious by including only statistically significant predictors of mortality and ensuring there were at least 15 outcomes per predictor in all models by subgroup. Moreover, as data on coronary angiogram was not available in the registry, we could not analyze which coronary artery would likely be the culprit for the worse prognosis in SMuRF-less patients. Additionally, as we attempted to include as many eligible patients as possible while ensuring the patients had a complete follow-up with the available death data, we could only evaluate short-term clinical outcomes (with the longest follow-up of 1-year mortality) among SMuRF-less patients in this study. Therefore, future studies not limited to patients with PCI, of bigger sample size, with data on coronary angiograms and longer-term outcomes will be useful to ascertain and build on our findings.

## Summary and Conclusion

The proportion of the SMuRF-less has been increasing in STEMI patients. In-hospital mortalities and 1-year cardiovascular mortality were significantly higher in SMuRF-less STEMI patients. However, this difference did not remain after adjusting for the significantly higher rates of anterior infarction, CPR, and Killip class observed in SMuRF-less. In contrast to Chinese and Malay patients, SMuRF-less patients of South Asian descent had a two-fold higher risk of in-hospital all-cause mortality even after adjusting for features of increased disease severity (higher Killip class, more CPR, and anterior infarction), suggesting the presence of so far unidentified CVD risk factors in this population that could provide an opportunity for intensifying secondary prevention in order to improve health outcomes in this high-risk patient group. Future studies need to be done to investigate potential unidentified AMI risk factors in STEMI patients.

## Data Availability Statement

The raw data supporting the conclusions of this article will be made available by the authors, without undue reservation.

## Ethics Statement

The studies involving human participants were reviewed and approved this study received an exemption review (SingHealth CIRB Reference No: 2016/2480) with a waiver for informed consent from patients as the study was based on de-identified data. Written informed consent for participation was not required for this study in accordance with the national legislation and the institutional requirements.

## Author Contributions

C-HS, JK, HB, and DH designed this study. HZ contributed to this study by obtaining and analyzing the data from SMIR. C-HS, JK, and DH contributed to interpreting the data. C-HS, JK, and HZ wrote the manuscripts. AH, DF, L-LF, PL, BL, PC, T-CY, JY, TC, MC, JT, and GF contributed to this study by providing constructive comments and insights. DH and HB supervised and provided a critical review of the manuscript. All authors contributed to the article and approved the submitted version.

## Funding

C-HS was supported by the National University of Singapore Yong Loo Lin School of Medicine's Junior Academic Faculty Scheme. JK was supported by the SingHealth Medical Student Talent Development Award (SMSTDA). AH was supported by Khoo Clinical Scholars Programme, Khoo Pilot Award (KP/2019/0034), Duke-NUS Medical School, and the National Medical Research Council (NMRC/CS_Seedfd/012/2018). DH was supported by the British Heart Foundation (CS/14/3/31002), the National Institute for Health Research, University College London Hospitals Biomedical Research Centre, Duke-National University of Singapore Medical School, and Singapore Ministry of Health's National Medical Research Council under its Clinician Scientist-Senior Investigator scheme (NMRC/CSA-SI/0011/2017), Centre Grant scheme (CG21APR1006), and the Collaborative Centre Grant scheme (NMRC/CG21APRC006). This study is based upon work from COST Action EU-CARDIOPROTECTION CA16225, supported by COST (European Cooperation in Science and Technology).

## Conflict of Interest

The authors declare that the research was conducted in the absence of any commercial or financial relationships that could be construed as a potential conflict of interest.

## Publisher's Note

All claims expressed in this article are solely those of the authors and do not necessarily represent those of their affiliated organizations, or those of the publisher, the editors and the reviewers. Any product that may be evaluated in this article, or claim that may be made by its manufacturer, is not guaranteed or endorsed by the publisher.
